# Cost-effectiveness analysis of the use of immunotherapy in metastatic solid tumours in Austria by applying the ESMO-Magnitude of Clinical Benefit Scale (ESMO-MCBS) version 1.1

**DOI:** 10.1016/j.esmoop.2021.100198

**Published:** 2021-06-25

**Authors:** M. Pichler, J. Steyrer

**Affiliations:** 1 Division of Oncology, Department of Internal Medicine, Medical University of Graz, Graz, Austria; 2 Interdisciplinary Institute for Management and Organizational Behaviour, Vienna University of Economics and Business, Wien, Austria

**Keywords:** immunotherapy, costs, ESMO-MCBS, Austria

## Abstract

**Background:**

Immune checkpoint inhibitors (ICIs) treatment is a breakthrough in managing metastatic solid tumours, but its use is associated with a high financial burden for public health care systems. Validated tools such as the European Society for Medical Oncology-Magnitude of Clinical Benefit Scale (ESMO-MCBS) are frameworks that might help to better assess the clinical benefit of these novel innovative cancer drugs.

**Methods:**

Here, we systematically analysed the number of European Medicines Agency-approved ICIs labels with an ESMO-MCBS grade <4 and the impact of the ICIs on incremental costs, gain of life years (LYs), quality-adjusted life years (QALYs) and the incremental cost-effectiveness ratio in the Austrian population.

**Results:**

Of 23 ICIs treatment settings, we identified three clinical scenarios in metastatic solid cancers with an ESMO-MCBS grade <4 with no otherwise approved alternatives. In triple-negative breast cancer (TNBC), the addition of first-line atezolizumab increased QALYs by 0.33 compared with nab-paclitaxel only, with an incremental cost per QALY of €143 853. In small-cell lung cancer (SCLC), the addition of first-line atezolizumab increased the QALY by 0.09, with an incremental cost per QALY of €373 256, and the addition of first-line durvalumab increased the QALYs by 0.11, with an incremental cost per QALY of €589 527.

**Conclusions:**

Overall, most of the approved ICIs carry significant clinical benefit (≥4). Although TNBC and SCLC are challenging treatment scenarios, currently approved ICIs with an ESMO-MCBS grade <4 substantially increase the cost of medical treatment, and under a willingness-to-pay threshold of €100 000, they do not have a cost-effective comparative benefit.

## Highlights

• Most European Medicines Agency-approved ICIs have an ESMO-MCBS with significant clinical benefit.
• In hard-to-treat types of cancer such as TNBC or SCLC, the ESMO-MCBS is <4.
• Under a willingness-to-pay threshold of €100.000, they do not have a cost-effective comparative benefit in Austria.

## Introduction

Immune checkpoint inhibition through monoclonal antibodies has revolutionized treatment across multiple types of cancer, enabling oncologists to envision potentially long-term disease stabilization approaches even in metastatic solid tumours.^1^ Although this advance in efficacy is encouraging, in addition to serious side-effects of these drugs, the financial burden (or financial toxicity) of immune checkpoint inhibitors (ICIs) clearly shows increasing expenditures, with annual treatment costs exceeding €100 000 per patient.^2^ In addition, the reported efficacy of ICIs varies substantially between different types of cancer. While the introduction of ICIs has tremendously increased the 5-year overall survival (OS) rate of patients with certain types of cancer (e.g. metastatic melanoma patients now carry a 5-year OS rate of 52%),^3^ other ICIs approvals are based solely on rather small clinical benefits (e.g. for small-cell lung cancer (SCLC), an improvement in OS of ∼2 months led to drug approval).^4^ Given the high economic burden of novel cancer drugs, especially ICIs, different cancer organizations, health care providers and insurance companies have gained more interest in quantitatively assessing the clinical benefit of these novel cancer drugs.^5^ To date, frameworks to evaluate novel innovative cancer drugs have been developed by several meaningful international cancer societies, including the American Society of Clinical Oncology (ASCO) Value Framework (AVF) and the European Society for Medical Oncology-Magnitude of Clinical Benefit Scale (ESMO-MCBS).^6^^,^^7^

The ESMO-MCBS was originally launched in 2015^7^ and revised in 2017 to the ESMO-MCBS version 1.1.^8^ The ESMO scale aims to provide a globally validated and rational stratification tool for innovative, cost-intensive cancer drugs, with the mission of ‘accountability for reasonableness’, which incorporates extensive field testing and the peer review of results for ‘reasonableness’.^7^ As value is based on considerations of the magnitude of clinical benefit as well as costs and confronted with the challenges of understanding the actual magnitude of the clinical benefit, the ESMO-MCBS was developed as a validated and reproducible scale that is applicable across the full range of solid tumours in oncology.^7^ The scoring criteria of the ESMO-MCBS comprise five different evaluation forms depending on the clinical scenario to evaluate, where information on OS, progression-free survival (PFS), disease-free survival, the hazard ratio, the response rate, quality of life, prognosis of the condition and toxicity is considered and rated.^8^ In the noncurative setting, treatments with an ESMO-MCBS grade of 5 and 4 are considered to provide substantial clinical benefit, whereas treatments with a grade <4 are not.^8^ In present and future public health policy, this rating might become more important to ensure access to these innovative but expensive cancer drugs for the majority of patients. In this study, we aimed to systematically analyse publicly available ESMO-MCBS information with a focus on European Medicines Agency (EMA)-approved ICIs for metastatic solid tumours and describe the theoretical impact of a threshold of ESMO-MCBS grade <4 on the availability, health care outcome and cost-effectiveness of these cancer drugs for Austrian cancer patients.

## Materials and methods

First, we defined the evaluation criteria for the investigated studies on the background of the following points based on publicly available information at the cut-off date of 31 December 2020: (i) all ICIs approved by the EMA; (ii) a publicly available ESMO-MCBS grade from the ESMO-MCBS Scorecard (https://www.esmo.org/guidelines/esmo-mcbs/esmo-mcbs-scorecards); (iii) inclusion of studies covering only metastatic solid cancers, namely, bladder, kidney, lung, head and neck, breast, gastrointestinal and liver cancers; and (iv) exclusion of haematological cancers and skin cancers (melanoma and Merkel cell carcinoma). ICI approvals based on studies with an ESMO-MCBS grade <4 were further investigated for cost-effectiveness as follows. In addition to the ESMO-MCBS, we cross-validated the ICIs with ESMO-MCBS <4 (i.e. low clinical benefit) using the ASCO AVF. Recently, the threshold of clinical benefit was published for the AVF. AVF scores <40 indicate low clinical benefit, and scores >45 indicate substantial clinical benefit (https://www.asco.org/sites/new-www.asco.org/files/content-files/advocacy-and-policy/documents/2016-May-Updated-Value-Framework-FAQ.pdf).

Costs were assessed within the context of the Austrian health care system and included only direct medical drug costs for ICIs. Accordingly, nonmedical direct costs [complication management, costs of comedications, intravenous drug administration, doctor consultation visits, additional blood tests and testing for the programmed death-ligand 1 (PD-L1) status] and indirect costs [loss of economic output due to days missed from work (morbidity costs) and premature death (mortality costs)] were not taken into account. Incremental drug (e.g. ICIs) costs were calculated as the difference between the total prices of treatment under the addition of ICIs compared with those of standard therapy. For each trial, incremental cancer drug costs were calculated using the available average wholesale price (AWSP or ‘Fabriksabgabepreis’) derived from the Austrian ‘Warenverzeichnis’ and were cross-checked in the local hospital pharmacy. We used the registrational trial’s reported dosage protocols and median administered doses (assuming an average patient with 70-kg body weight). For atezolizumab, the costs were based on a dose of 840 mg every 2 weeks (€3360 per dose) or 1200 mg every 3 weeks (€47 992 per dose), and for durvalumab, the costs were based on a dose of 1500 mg (€9264 per dose). For the calculations of economic variables, we made the assumption of a model with three health states, namely, PFS, progressive disease (PD) and death.

For all treatment scenarios, we calculated the gain of life years (LYs), the quality-adjusted life year (QALY) and the incremental cost-effectiveness ratio (ICER). To calculate the gain of LYs, we calculated the difference in the median OS between the ICI-treated group and the standard of care group. Basically, the QALY is a generic measure of disease burden and includes both the quality and quantity of life lived.^9^ To determine QALYs, we multiplied the health utility (weight) value associated with a given state of health by the years lived in that state. For instance, a year of life lived in perfect health is worth 1 QALY [1 year of life years (LYs) × 1 utility value]. The health utilities are numerical values that represent an individual’s preferences for different health-related outcomes, ranging from 0 (representing a state of death) to 1 (representing a state of perfect health).^9^ Previous studies on metastatic breast cancer reported health utility values of 0.715 for PFS and 0.443 for PD.^10^ For SCLC, the reported health utility values were 0.673 for PFS and 0.473 for PD.^11^ Given these assumptions, we calculated the QALY as follows: [median PFS (in years) × utility value PFS] plus [median OS minus median PFS (in years) × utility value PD]. The gain of QALY was calculated as the difference between the QALY of the ICI group and the QALY of the standard of care group. The ICER was calculated by the incremental costs divided by the gained LYs or the gained QALY.

In addition to our calculations described above based on median PFS/OS times, we developed a partitioned survival model to simulate the clinical outcome and economic costs for one ICI with ESMO-MCBS <4, according to data of the CASPIAN trial.^12^ This trial compared the conventional chemotherapy only with chemotherapy plus the ICI durvalumab. The chemotherapy only group received between four and six (we averaged five cycles in the model) cycles of platinum–etoposide. Patients presenting disease progression or unacceptable adverse reactions received a second-line treatment with topotecan. Our model structure included the following three states: PFS, PD and death. Time-to-event tables were used for the simulation and assignment to a certain state. The primary outcomes of our model simulation included total cost, QALYs and LYs. The ICER was also calculated by the software.

Clinical efficacy data for the first-line treatments, including the Kaplan–Meier curves of PFS and OS, were derived from the phase III CASPIAN study.^12^ The GetData Graph Digitizer (version 2.26; http://www.getdata-graph-digitizer.com/download.php) was used to extract the PFS and OS probabilities from the PFS and OS curves of each treatment group as previously described.^13^ The individual patient data of each Kaplan–Meier curve was reconstructed and survival tables were used to fit the data. Distribution functions were based on Weibull distribution [S(t) = exp(−ltg)] according to the suggestions of the TreeAge Pro software. For the partitioned survival analysis-based cost-effectiveness analysis, we calculated for the chemotherapy only group five cycles of chemotherapy (according to the CASPIAN study, 75% received carboplatin and 25% received cisplatin).^12^ For the ICIs group durvalumab was calculated for four cycles at a dose of 1500 mg every 21 days, followed by continued use of durvalumab every 4 weeks for a median of seven doses. We assumed for a body surface of 1.89 m^2^ that a one-cycle dose of the chemotherapy drugs included cisplatin (80 mg/m^2^; costs per cycle: €68.4), carboplatin area under the curve of 5 mg/ml/min (costs per cycle: €136), etoposide 90 mg/m^2^ on days 1-3 of each cycle (costs per cycle: €57.9), durvalumab (costs per cycle: €9264) and for the second-line treatment upon progression topotecan 1.5 mg/m^2^/day on days 1-5 for each cycle (costs per cycle: €1217). Patients in the immunotherapy group received four cycles of chemotherapy (combined costs per cycle: €9441). Analyses were conducted using a partitioned survival model (Supplementary Figure S1, available at https://doi.org/10.1016/j.esmoop.2021.100198) constructed with the TreeAge Pro 2021 software (TreeAge, Williamstown, Massachusetts).

Potential patient numbers were retrieved from the publicly available central registry of the Austrian Bureau of Statistics (Statistik Austria).

## Results

### EMA-approved ICIs and corresponding ESMO-MCBS grade

Overall, there are currently six EMA-approved ICIs available for different types of metastatic solid tumours. These approvals include the three PD-L1 inhibitors atezolizumab, avelumab and durvalumab; the two PD1 inhibitors nivolumab and pembrolizumab and the cytotoxic T-lymphocyte-associated protein 4 inhibitor ipilimumab (Table 1). As shown in Table 1, a total of 23 approved treatment settings for these ICIs were identified in the publicly available ESMO-MCBS Scorecard. Of the 23 different treatment settings, 19 were excluded from further cost-effectiveness evaluations: 15 had an ESMO-MCBS grade of ≥4, two bladder cancer approvals had not yet been graded (i.e. MCBS not available) and two treatment settings in non-SCLC (atezolizumab in combination with bevacizumab/chemotherapy^14^ and ipilimumab in combination with nivolumab/chemotherapy^15^) have an alternative ICI-containing treatment approach with an ESMO-MCBS grade ≥4 (pembrolizumab in combination with chemotherapy^16^). Thus, for further cost-effectiveness analyses, we selected three ICI-containing clinical scenarios in metastatic solid tumours identified with an ESMO-MCBS grade <4 (i.e. not clinically significant according to the ESMO definition): (i) in triple-negative breast cancer (TNBC), the first-line atezolizumab approval (based on the data of the IMpassion130 trial^17^) and (ii) in SCLC, the first-line atezolizumab approval (based on the data of the IMpower133 trial^4^) and (iii) the first-line durvalumab approval (based on the data of the CASPIAN trial^12^). In addition, we manually calculated an ASCO-based AVF score <40 for all these three treatment scenarios, indirectly confirming the low clinical benefit in another scoring framework.

**Table 1:** Summary of EMA-approved immune checkpoint inhibitors in metastatic solid tumours, the corresponding publicly available ESMO-MCBS (https://www.esmo.org/guidelines/esmo-mcbs/esmo-mcbs-scorecards) and potential immune checkpoint inhibitor alternatives with a higher ESMO-MCBS

Drug name	Tumour type	ESMO-MCBS	Alternatives
Atezolizumab (Tecentriq)	Bladder cancer first line	n.a.	Yes
	Bladder cancer second line	n.a.	Yes
	Non-small-cell lung cancer second Line	5	Yes
	Non-small-cell lung cancer first line (combination with chemotherapy and bevacizumab)	3	Yes
	Small-cell lung cancer first line	3	No
	Triple-negative breast cancer	3	No
	Hepatocellular carcinoma	5	No
Avelumab (Bavencio)	Renal cell carcinoma first line	3	Yes
Durvalumab (Imfinzi)	Stage III non-small-cell lung cancer	4	No
	Small-cell lung cancer	3	No
Nivolumab (Optivo)	Non-small-cell lung cancer second line	5	Yes
	Renal cell carcinoma second line	4	No
	Renal cell carcinoma first line	4	Yes
	Head and neck cancer second line	5	Yes
	Oesophagus cancer second line	4	No
Pembrolizumab (Keytruda)	Non-small-cell lung cancer second line	5	Yes
	Non-small-cell lung cancer first line (combination chemotherapy)	4	Yes
	Non-small-cell lung cancer first line PD-L1 >50%	5	No
	Renal cell carcinoma first line	4	Yes
	Head and neck cancer first line	4-5	No
	Head and neck cancer second line	4	Yes
Ipilimumab (Yervoy)	Renal cell carcinoma first line (combination with nivolumab)	4	Yes
	Non-small-cell lung cancer first line (combination with nivolumab and chemotherapy)	2	Yes

EMA, European Medicines Agency; ESMO, European Society for Medical Oncology; MCBS, Magnitude of Clinical Benefit Scale; n.a., not available.

### Cost-effectiveness calculation of ICIs with ESMO-MCBS <4

To calculate the incremental drug (e.g. ICIs) costs compared with standard of care treatment, we analysed the costs per dose in the context of the clinical trial data. Table 2 shows a study summary of these three treatment settings. In TNBC patients, the median PFS observed with atezolizumab–nab-paclitaxel was 7.5 months compared with 5.0 months with placebo–nab-paclitaxel (difference 2.5 months). The most recently updated clinical data from PD-L1-positive TNBC patients showed a median OS duration of 25.4 months in the atezolizumab–nab-paclitaxel group and 17.9 months in the placebo–nab-paclitaxel group.^17^ The incremental drug costs in Austria were €47 040 per patient, assuming a median treatment duration of seven cycles (14 doses of 840 mg atezolizumab every 2 weeks).^17^ Cost-effectiveness analysis of the use of atezolizumab in TNBC patients in Austria led to an average gain of 0.625 LYs [difference in the median OS (months) in the atezolizumab–nab-paclitaxel group and median OS (months) in the nab-paclitaxel only group divided by 12], a QALY of 1.10 in the atezolizumab–nab-paclitaxel group and a QALY of 0.77 in the chemotherapy only group, resulting in a gain of QALY of 0.33 per person. These benefits were achieved at an incremental cost of €47 040 per person (as calculated earlier). As a result, the addition of atezolizumab to nab-paclitaxel was associated with an ICER of €75 264 per LY gained and €143 853 per QALY gained.

**Table 2:** Clinical benefit scores and incremental cancer drug costs for immune checkpoint inhibitors across different settings in metastatic solid tumours with an ESMO-MCBS of <4

Setting/trials name	Experimental/control arm	ESMO-MCBS	Treatment doses, median	Costs per dose/incremental costs
TNBC/IMpassion130	Atezolizumab plus nab-paclitaxel/nab-paclitaxel	3	14	€3360/€47 040
SCLC/IMpower133	Atezolizumab plus carboplatin-etoposide/carboplatin-etoposide	3	7	€47 992/€33 593
SCLC/CASPAN	Durvalumab plus platinum-etoposide/platinum-etoposide	3	7	€9264/€64 848

ESMO, European Society for Medical Oncology; MCBS, Magnitude of Clinical Benefit Scale; SCLC, small-cell lung cancer; TNBC, triple-negative breast cancer.

Concerning atezolizumab in SCLC patients, according to the IMpower133 phase III study,^18^ the median PFS was 5.2 months in the atezolizumab/chemotherapy group and 4.3 months in the chemotherapy only group (difference 0.9 months), and the median OS was 12.3 months in the atezolizumab group and 10.3 months in the placebo group (difference = 2 months).^18^ The incremental drug costs in Austria were €33 593 per patient, assuming a median number of atezolizumab doses of seven (1200 mg atezolizumab per dose), as previously reported.^4^ Cost-effectiveness analysis using data from the IMpower133 study showed an average gain of 0.17 LYs (difference in OS in the atezolizumab group and OS in the standard of care group divided by 12), a QALY of 0.57 in the atezolizumab/chemotherapy group and a QALY of 0.48 in the chemotherapy only group, resulting in a gain of QALY of 0.09 per person. These benefits were achieved at an incremental cost of €33 593 in Austria. As a result, the addition of atezolizumab to chemotherapy in SCLC was associated with an ICER of €197 606 per LY gained and €373 256 per QALY gained.

Regarding durvalumab in SCLC patients, the median PFS was 5.1 months with durvalumab plus chemotherapy versus 5.4 months with chemotherapy only, and the median OS was 13.0 months with durvalumab plus chemotherapy versus 10.3 months with chemotherapy only (difference 2.7 months).^12^ The incremental drug costs with durvalumab were €64 848 per patient, assuming a median number of durvalumab doses of seven, as previously described.^12^ Cost-effectiveness analysis of the CASPIAN study showed an average gain of 0.23 LYs (difference in OS in the durvalumab group and OS in the chemotherapy only group divided by 12), a QALY of 0.60 in the durvalumab/chemotherapy group and a QALY of 0.49 in the chemotherapy only group, resulting in a gain of QALY of 0.11 per person. These benefits were achieved at an incremental cost of €64 848 per patient. As a result, the addition of durvalumab to chemotherapy in SCLC was associated with a deterministic base-case ICER of €281 948 per LY gained and €589 527 per QALY gained.

**Figure 1:**
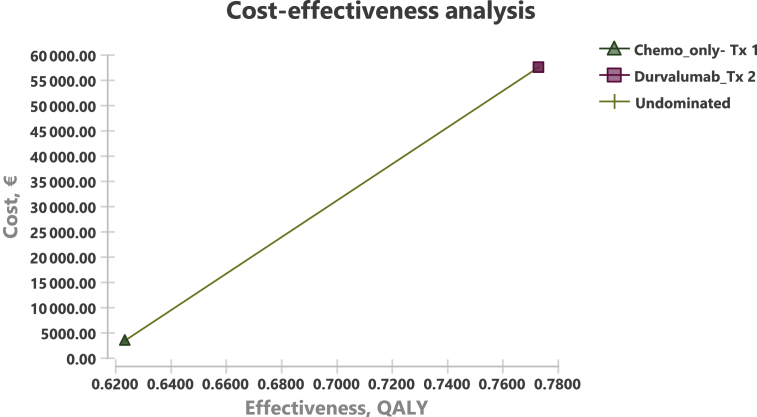
Cost-effectiveness analysis comparing chemotherapy only with addition of Durvalumab. Incremental costs per QALY are shown (derived from TreeAge Pro Software package). QALY, quality-adjusted life year.

Applying partitioned survival analysis-based cost-effectiveness calculations (Supplementary Figures S2 and S3, available at https://doi.org/10.1016/j.esmoop.2021.100198), we found in the durvalumab/chemotherapy group incremental drug costs of €50 331 per patient (€53 765 per patient versus €343 444 per patient in the chemotherapy only group), and a gain of QALY of 0.15 per person (0.77 QALYs in the durvalumab/chemotherapy group and 0.62 QALYs in the chemotherapy only group). Overall, durvalumab/chemotherapy as the first-line treatment for SCLC had an ICER of €336 534 per QALY compared with chemotherapy only treatment (Figure 1).

### Potential cost-saving effects in the Austrian population

Finally, to calculate the potential cost-saving effect based on the assumption that the entire Austrian target population of patients with metastatic solid tumours of ESMO-MCBS grade <4 will be not treated with ICIs (as discussed earlier), we next estimated the putative size of the target population (i.e. patients with metastatic TNBC and extensive SCLC). The incidence rate of breast cancer has been relatively stable within the last 10 years, with ∼5500 newly diagnosed breast cancer cases in Austria (Supplementary Figure S4, available at https://doi.org/10.1016/j.esmoop.2021.100198). Of these, ∼15% of cases were diagnosed as the TNBC subtype (*n* = 825). Taking into account that ~5%^19^ of cases are diagnosed as metastatic TNBC at the time of diagnosis (*n* = 41) and up to 40%^20^ of primarily localized stage I-III TNBC cases (*n* = 784) recur within 5 years (*n* = 314), the number of metastatic TNBC cases per year is estimated to be ∼355. According to the atezolizumab registrational trial IMpassion130,^17^ 41% of TNBC patients are PD-L1 ≥1% positive, a requirement for the administration of this drug. Thus, we estimated for our cost calculation model the number of potentially metastatic PD-L1-positive TNBC patients to be ∼146 per year in Austria. Of these, we estimated the proportion of immunotherapy-ineligible patients (older age, comorbidities, performance status and comedications) in this rather young patient cohort to be ∼10%, resulting in an absolute number of 130 TNBC patients per year.

The lung cancer incidence in Austria in 2017 was 4676 (2739 males and 1937 females, Supplementary Figure S5, available at https://doi.org/10.1016/j.esmoop.2021.100198), and ∼15%^21^ of the patients (*n* = 700) were diagnosed with SCLC. Of these SCLC patients, ∼70%^22^ were diagnosed primarily with extensive SCLC (*n* = 490) and were considered for upfront palliative combination therapy with platinum/etoposide plus atezolizumab or durvalumab. However, as a substantial number of SCLC patients carry relevant comorbidities, are in an advanced disease state, exhibit a reduced performance status and are aged >75 years, many of them fail to receive this standard regimen.^23^ Although systematic data on the percentage of combination therapy-ineligible patients in Austria are not available, in our experience, we estimate that ∼50% of patients with extensive SCLC will not qualify for combination immunochemotherapy. Thus, we used an estimated patient number of 350 per year with extensive SCLC who were eligible for combination therapy in Austria.

The savings potential for ruling out drugs with an ESMO-MCBS grade <4 was extrapolated over the yearly incidence and potential size of the target population of TNBC and SCLC patients in Austria (according to the formula AWSP multiplied by cases per year). By applying the ESMO-MCBS, a savings benefit from €6.1 million (for atezolizumab in TNBC) up to €11.7 million (atezolizumab in SCLC) or €22.7 million (durvalumab in SCLC) can be reached.

## Discussion

The ESMO-MCBS was introduced as a tool to aid in clinical decisions and to identify novel cancer drugs with a graduation depending on the robustness, availability and relevance of clinical trial-generated data.^7^ Thus, a grade ≥4 has been defined as a treatment setting with significant clinical benefit.^7^ In our analysis, after applying the criteria defined in the ‘Methods’ section we found that 3 of 23 (13%) currently approved treatment approaches for metastatic solid tumours had no significant clinical benefit (ESMO-MCBS grade <4). First, this is good news, as for the majority of ICI treatment schedules, solid and high-quality clinical results led to the definition of ‘significant clinical benefit’. This underlines the impact that these new drugs have on improving meaningful clinical endpoints, improving quality of life, or reducing toxicity compared with classic cytotoxic treatment schedules. By contrast, the three treatment scenarios with an ESMO-MCBS grade <4 are challenging types of cancer. For instance, metastatic TNBC commonly affects premenopausal young women and is associated with an aggressive clinical course, a high disease burden, high morbidity and a poor prognosis due to a significant lack of other treatment options.^24^ Unfortunately, disseminated SCLC patients have a median OS of 6-10 months, with no new cancer drug approved within the last 20 years.^23^

According to our analysis, atezolizumab combined with nab-paclitaxel for metastatic TNBC is not cost-effective in Austria from the perspective of different definitions: The NICE (UK) threshold for what is considered an acceptable value ranges between GB£20 000 and GB£30 000 (or ∼US$30 000–45 000), and the American College of Cardiology/American Heart association used the WHO benchmark that is based on a country’s gross domestic product (GDP) per capita.^25^ Services that exceed three times GDP per capita are viewed as economically unattractive (based on the 2020 Austrian GDP, which is approximately >€150 000 per QALY; https://www.worlddata.info/europe/austria/economy.php). In our study, the incremental cost of atezolizumab for TNBC patients was €47 040, the incremental QALY was 0.11 and the ICER was €143 853 per QALY. The first cost-effectiveness analysis of atezolizumab in TNBC patients was performed by Weng et al.,^13^ who reported that for the United States, the ICER value was US$229 359.88 per QALY gained for the PD-L1-positive population, and in China, the ICER value was US$72 971.88 per QALY gained. The authors concluded that the use of atezolizumab was not cost-effective at the willingness-to-pay thresholds of US$150 000 per QALY in the United States and US$29 383 per QALY in China. Li et al.^26^ reported an ICER of $361 218 per QALY gained in the PD-L1-positive subgroup and concluded again that atezolizumab is not cost-effective in these patient populations. The latest economic analysis was performed by Phua and colleagues,^27^ who reported an additional gain of 0.361 QALYs (0.636 LYs) at an ICER of US$324 550 per QALY gained. In summary, all studies evaluating atezolizumab as a first-line treatment for TNBC describe poor cost-effectiveness in different countries and continents, underlining the necessity for improved price models to ensure broad access to this new treatment approach.

According to our analysis, atezolizumab combined with platinum/etoposide for extensive SCLC is not cost-effective in Austria from the perspective of the aforesaid definition. The incremental cost per patient was €33 593, the incremental QALY was 0.09 and the ICER was €373 253 per QALY. In line with our findings, the first report by Zhou and colleagues^11^ showed that treatment with atezolizumab plus chemotherapy was estimated to increase costs by US$52 881 compared with chemotherapy alone, with a gain of 0.10 QALYs, leading to an incremental cost-effective ratio of US$528 810 per QALY. Similar findings were reported in a cost-effectiveness analysis in a study from China, where the authors concluded that atezolizumab combination therapy was not more cost-effective than chemotherapy alone at a world trade product threshold of US$25 929 per QALY.^28^

According to our analysis, durvalumab combined with platinum/etoposide for extensive SCLC is not cost-effective in Austria from the perspective of the definitions in the United Kingdom, United States and WHO. The incremental cost per patient was US$64 848, the incremental QALY was 0.11 and the ICER was €589 527 per QALY. Although a comparison with other health care systems is difficult, Zhang and colleagues^29^ applied a partitioned survival model and calculated the price of the same treatment setting on the basis of the Medicare drug average sales price from the US Centers for Medicare & Medicaid. They reported an incremental cost per patient of US$78 019, while the incremental QALY was 0.220, and the ICER was US$355 448.86 per QALY.^29^ Expanding this view to the Chinese health care system, Liu and Kang^30^ reported that durvalumab plus chemotherapy yielded additional 0.25 QALYs, with incremental costs of US$76 354, resulting in an ICER of US$302 051 per QALY compared with chemotherapy alone. Even when patient assistant program was available, the ICER was US$192 591 per QALY, leading the authors to conclude that durvalumab in SCLC first-line treatment will be unlikely to be cost-effective in China. Similar findings were reported in our partitioned survival analysis done for the CASPIAN trial: the durvalumab/chemotherapy group incremental drug costs of €50 331 per patient, and a gain of QALY of 0.15 per person, with an ICER of €336 534 per QALY compared with chemotherapy only treatment. Vogler et al.^31^ compared the prize of 31 cancer drugs in 18 different countries including 16 different European countries. The difference of a drug official undiscounted price between the highest priced country and the lowest priced country varied between 28% and 388%. Similar findings showing a high degree of cancer drug cost variation were reported in a survey of 15 European countries.^32^ Thus, our approach and findings are applicable to other European countries, though it strongly depends on the country-specific drug prize and (unreported) discounts.

In our model, we determined the cost and utility of first-line durvalumab for only seven doses, as this was the median dosage reported in the CASPIAN trial. Nevertheless, Zhang et al.^29^ analysed cost-effectiveness not only for seven doses but also for 1 year, 2 years and a lifetime to avoid the influence of the duration of durvalumab, and their results indicated that the durvalumab plus chemotherapy regimen is not economical in all evaluated situations. The authors also concluded that durvalumab combined with chemotherapy was not a cost-effective approach, consistent with the results of our study on the Austrian system.

The findings for both atezolizumab and durvalumab in extensive SCLC and their disappointing cost-effectiveness are in contrast to those for pembrolizumab in the first-line treatment of NSCLC, where the PD1 inhibitor pembrolizumab with or without chemotherapy has been suggested to be a cost-effective option as a first-line treatment for metastatic NSCLC expressing high levels of PD-L1.^33^ Generally, predictive biomarkers might help to enrich patients with a high probability of responding and therefore decrease the ICER and increase the gain of QALYs. Nevertheless, according to the IMpower133 trial, in SCLC, PD-L1 is either not frequently expressed or its tumour mutational burden (another hypothetical predictive biomarker) cannot be successfully validated as useful.^4^

In its most recently updated report for 2020-2023, the Institute of Clinical and Economic Review (ICER) recommended cost-effectiveness thresholds with a maximum between US$100 000 and US$150 000 per QALY (https://icer.org/our-approach/methods-process/value-assessment-framework). None of our scenarios fulfilled these criteria, and cost-effectiveness for the ESMO-MCBS grade <4 ICI treatment schedules in Austria has to be refused. Finally, the low clinical benefit of these three treatment scenarios could also be confirmed in the ASCO-based AVL framework (AVL score <40). In addition to the ESMO and ASCO framework, the NCCN has established an NCCN Framework for Resource Stratification of NCCN Guidelines (NCCN Framework) tool, which defines appropriate treatment pathways based on available resources—Basic, Core, Enhanced, and NCCN Guidelines (https://www.nccn.org/guidelines/nccn-framework-for-resource-stratification-of-nccn-guidelines). This framework might be another resource for different countries in decision making to select cost-efficient treatment schedules.

Our study is not without limitation. Our analyses were based on three clinical trials in which patients were generally younger and experienced a longer survival time than those in clinical real-world scenarios. Next, we used the whole sale price (Fabriksabgabepreis) to estimate the drug costs. Health care providers (hospitals) in Austria are contracting discounts, risk share agreements and financial caps with pharmaceutical companies, but the lack of publicly available sources reporting contracts poses challenges in the usage of the whole sale price for cost-effectiveness analyses. The real costs for ICIs will be quite lower than the price calculations of our analyses. By contrast, costs increase with the use of ICIs through the surveillance of side-effects, expanded laboratory tests to detect immune-related side-effects and hospitalization due to complication management and severe adverse events.

Despite the mentioned limitations, the results of these analyses suggest an ESMO-MCBS grade <4 to reflect high costs in the context of expected clinical benefit for the Austrian population.
