# Supplementary figures and images for: Cost-effectiveness analysis of the use of immunotherapy in metastatic solid tumours in Austria by applying the ESMO-Magnitude of Clinical Benefit Scale (ESMO-MCBS) version 1.1
Source: ESMO Open. 2021 Jun 25;6(4):100198. doi: 10.1016/j.esmoop.2021.100198 (PMC8253953; doi:10.1016/j.esmoop.2021.100198)

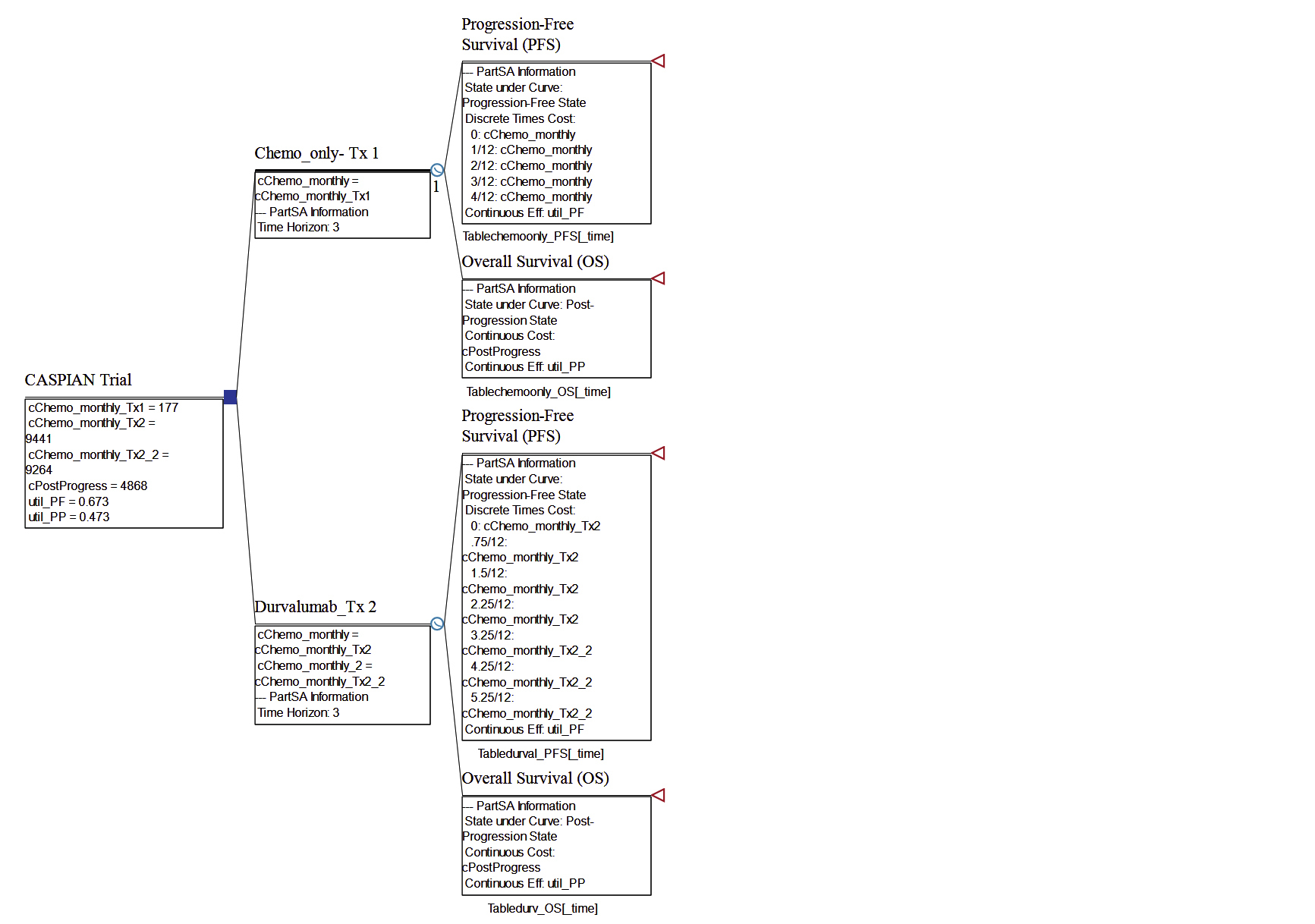

Supplement: Supplementary Figure S1 [file figs1.jpg]

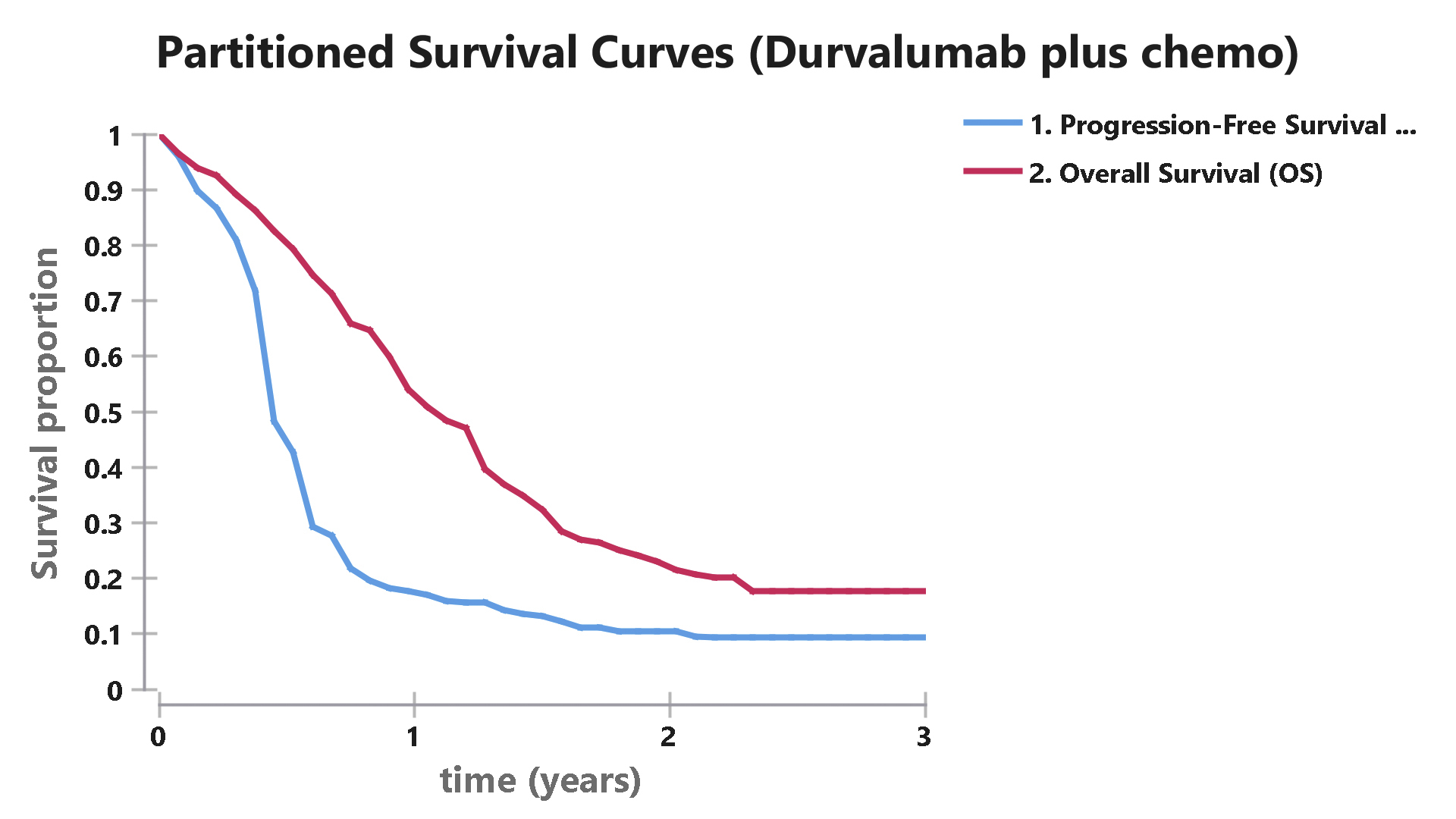

Supplement: Supplementary Figure S2 [file figs2.jpg]

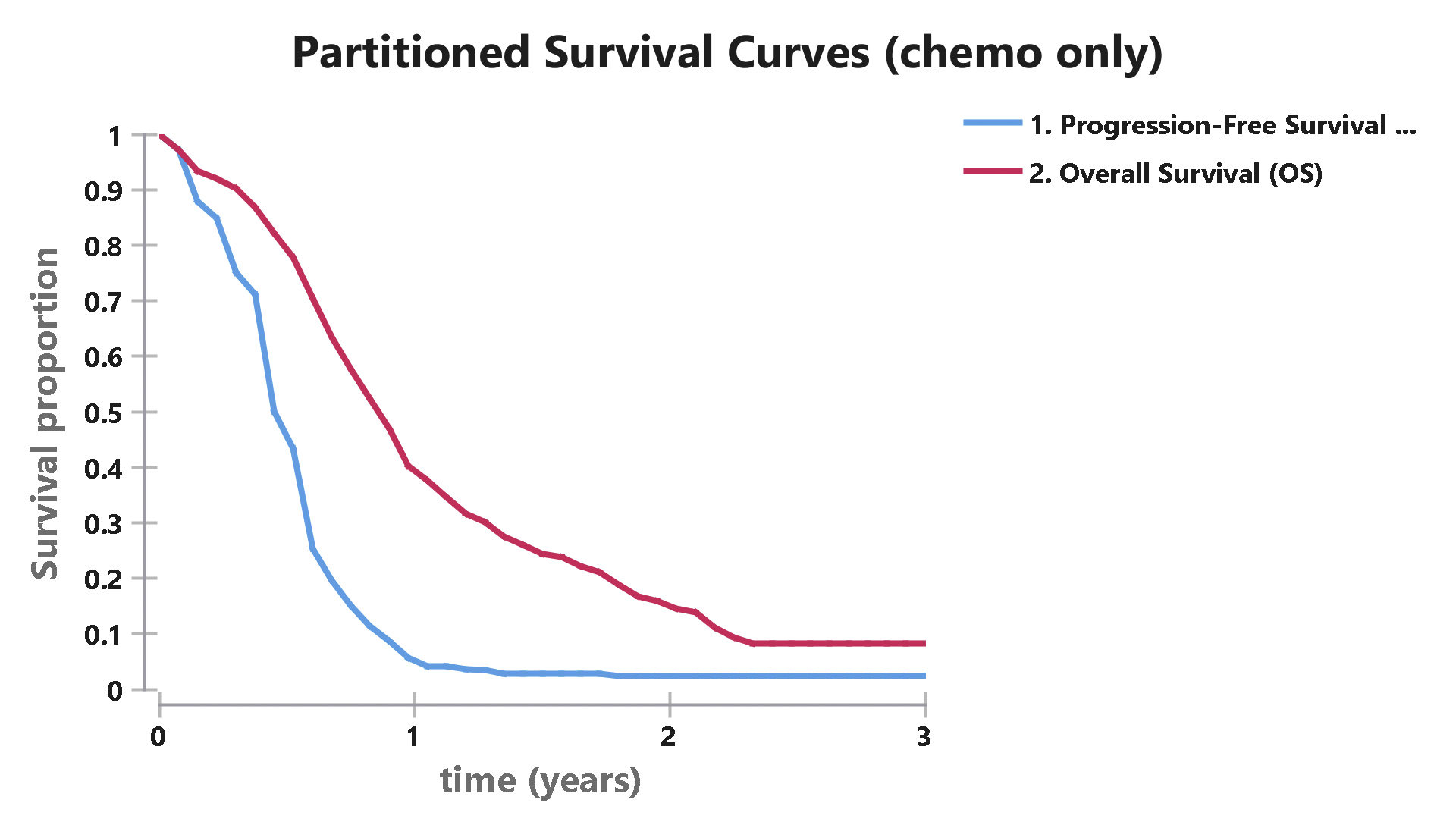

Supplement: Supplementary Figure S3 [file figs3.jpg]

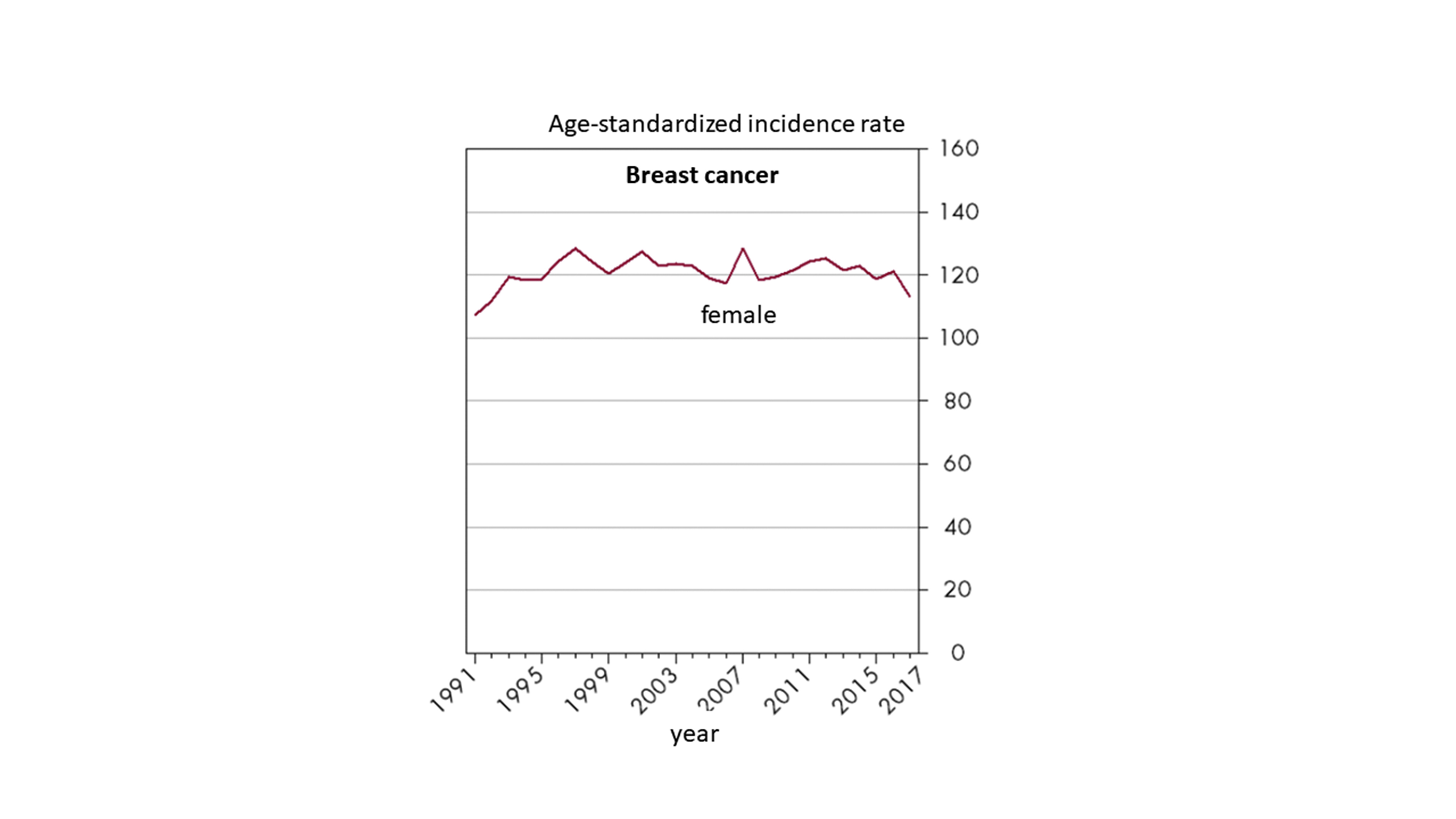

Supplement: Supplementary Figure S4 [file figs4.jpg]

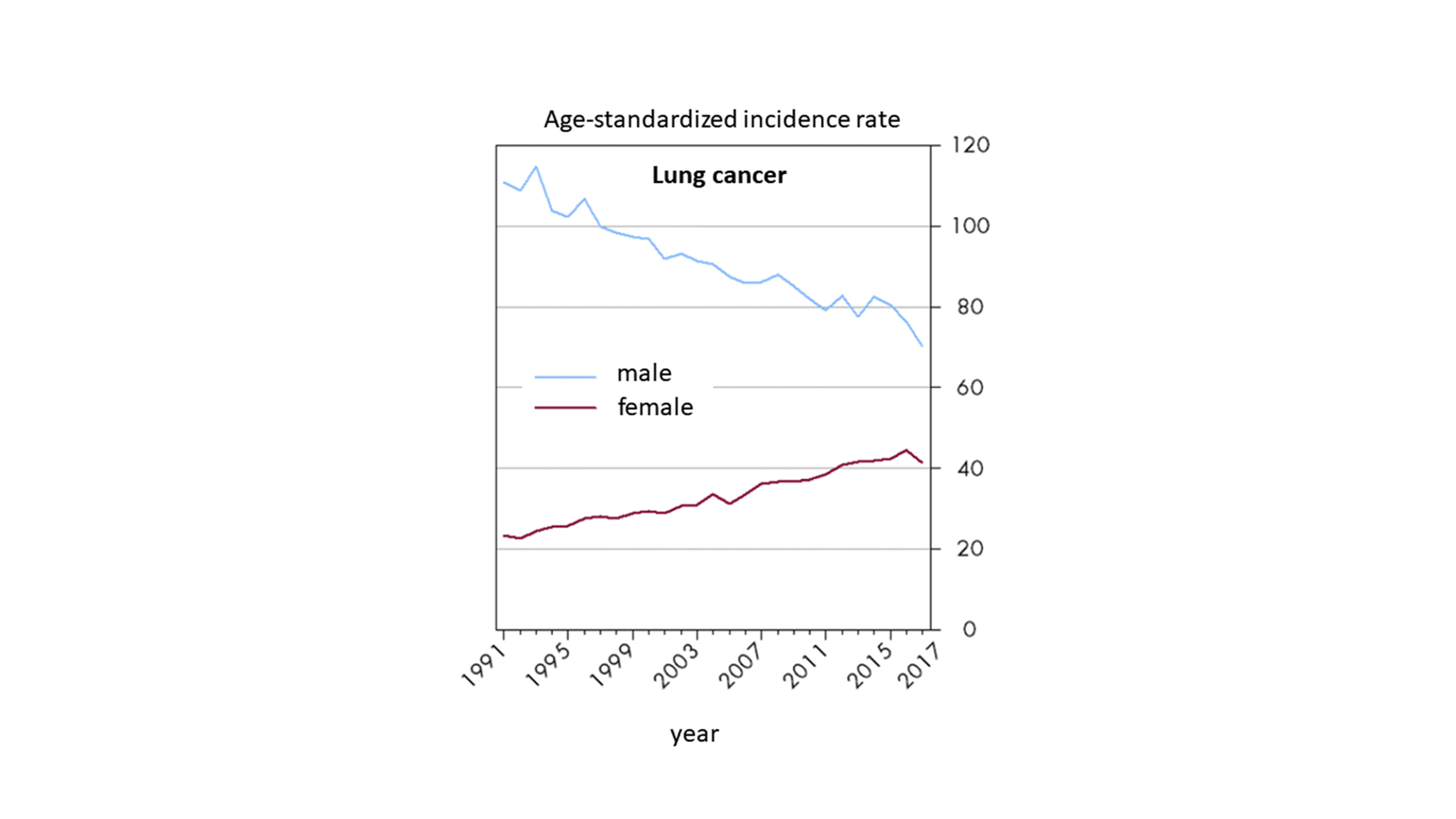

Supplement: Supplementary Figure S5 [file figs5.jpg]
