# Supplementary material for: Cost-effectiveness analysis of the use of immunotherapy in metastatic solid tumours in Austria by applying the ESMO-Magnitude of Clinical Benefit Scale (ESMO-MCBS) version 1.1
Source: ESMO Open. 2021 Jun 25;6(4):100198. doi: 10.1016/j.esmoop.2021.100198 (PMC8253953; doi:10.1016/j.esmoop.2021.100198)
Supplement: Supplementary file 2 [file mmc1.zip › mmc1/mmc1.odt]

Supplementary Figure S1. Partitioned survival analysis model used for cost-effectiveness analysis in TreeAge pro Software.

Supplementary Figure S2. Partitioned survival curves showing overall survival and progression-free survival curves derived from the CASPIAN trial for the group that received durvalumab/chemotherapy.

Supplementary Figure S3. Partitioned survival curves showing overall survival and progression-free survival curves derived from the CASPIAN trial for the group that received chemotherapy only.

Supplementary Figure S4. Age-standardized incidence rate of breast cancer over the last 30 years in Austria (courtesy Statistik Austria).

Supplementary Figure S5. Age-standardized incidence rate of lung cancer over the last 30 years in Austria (courtesy Statistik Austria).
